# Spironolactone promotes autophagy via inhibiting PI3K/AKT/mTOR signalling pathway and reduce adhesive capacity damage in podocytes under mechanical stress

**DOI:** 10.1042/BSR20160086

**Published:** 2016-07-08

**Authors:** Dong Li, Zhenyu Lu, Zhongwei Xu, Junya Ji, Zhenfeng Zheng, Shan Lin, Tiekun Yan

**Affiliations:** * Department of Nephrology, General Hospital of Tianjin Medical University, Tianjin, 300052, China; † Tianjin Precell Biotechnology Co., Ltd., Huayuan Industrial District, Tianjin, 300384, P.R. China; ‡ Central Laboratory, Logistics University of the Chinese People's Armed Police Force, Tianjin, 300309, China

**Keywords:** Atg5, diabetic nephropathy, integrin

## Abstract

Mechanical stress which would cause deleterious adhesive effects on podocytes is considered a major contributor to the early progress of diabetic nephropathy (DN). Our previous study has shown that spironolactone could ameliorate podocytic adhesive capacity in diabetic rats. Autophagy has been reported to have a protective role against renal injury. The present study investigated the underlying mechanisms by which spironolactone reduced adhesive capacity damage in podocytes under mechanical stress, focusing on the involvement of autophagy. Human conditional immortalized podocytes exposed to mechanical stress were treated with spironolactone, LY294002 or rapamycin for 48 h. The accumulation of LC3 puncta was detected by immunofluorescence staining. Podocyte expression of mineralocorticoid receptor (MR), integrin β1, LC3, Atg5, p85-PI3K, p-Akt, p-mTOR were detected by Western blotting. Podocyte adhesion to collagen type IV was also performed with spectrophotometry. Immunofluorescence staining showed that the normal level of autophagy was reduced in podocytes under mechanical stress. Decreased integrin β1, LC3, Atg5 and abnormal activation of the PI3K/Akt/mTOR pathway were also detected in podocytes under mechanical stress. Spironolactone up-regulated integrin β1, LC3, Atg5 expression, down-regulated p85-PI3K, p-Akt, p-mTOR expression and reduced podocytic adhesive capacity damage. Our data demonstrated that spironolactone inhibited mechanical-stress-induced podocytic adhesive capacity damage through blocking PI3K/Akt/mTOR pathway and restoring autophagy activity.

## INTRODUCTION

Diabetic nephropathy (DN) is a serious complication of diabetes and a major indication for dialysis and transplantation among the diabetic complications [1,2]. DN is characterized by podocyte loss due to its detachment from the glomerular basement membrane (GBM). Podocytes are terminally differentiated cells which have been regarded as the key target of harmful stimuli in renal disease especially under diabetic conditions [3]. Haemodynamic changes, characterized by increases in high intracapsular pressure, hyperfusion and hyperfiltration which would cause deleterious adhesive effects on podocytes are central in the early stage of DN [4,5]. Autophagy is a stress response involved in damaged protein and organelle degradation which has been reported to have a protective role against renal injury induced by aging [6], hypoxia [7] etc. Mechanical stress deriving from glomerular hypertension is one of the important damage factors underlying pathogenesis of DN. However, the relationship between mechanical stress and podocytic autophagy remains to be elucidated.

In recent years, clinical and experimental studies have demonstrated that aldosterone acts as a mediator in the pathogenesis of DN [8]. Moreover, the protein expression of mineralocorticoid receptor [MR; nuclear receptor subfamily 3, group C member 2 (NR3C2)] can be consistently observed in cultured podocyte which hence is one of the target cells for the deleterious effects of aldosterone [9]. Our previous study demonstrated that spironolactone, a nonselective MR blocker, could produce beneficial effects in streptozotocin-induced diabetic rats [10]. Clinical trials also suggest that addition of spironolactone to an angiotensin-converting enzyme inhibitor may further improve proteinuria in patients with DN [11]. Mannic et al. [12] showed that aldosterone could promote the phosphorylation of Akt by phosphatidylinositol 3-kinase (PI3K) in cardiomyocytes. In mammalian cells, PI3K/AKT/mTOR signalling pathway is a classic negative regulatory pathway for autophagy when cells are exposed to certain conditions, such as starvation, oxidative stress, infection and tumour suppression [13]. Thus, we hypothesize that spironolactone could promote autophagy via PI3K/AKT/mTOR signalling pathway in podocytes. Our present study was designed to determine the role of autophagy in podocytic adhesive capacity damage under mechanical stress and the underlying mechanisms of potential beneficial effects of spironolactone on podocytes.

## MATERIALS AND METHODS

### Cell culture and grouping

A conditionally immortalized human podocyte cell line (provided by Prof. Moin Saleem, Bristol Royal Hospital for Children, Bristol, UK) has been described in detail previously [14]. Podocytes were cultured in RPMI1640 medium supplemented with 10% heat-inactivated FBS, 100 U/ml penicillin and 100 mg/ml streptomycin in the presence of interferon-γ at 33°C in 5% CO_2_/95% air (permissive condition). To induce differentiation, we maintained podocytes at 37°C without IFN-γ (nonpermissive conditions) for 10–14 days. Then they were seeded in six-well silastic culture plates (Biopress plates, Flexcell International) and covered with 2 μg/cm^2^ human collagen type IV (Santa-Cruz Biotechnology) before applying mechanical stress. After that the cells were divided into the following groups: Group CON, plated cells were statically cultured for 48 h to serve as a control; Group STS, cells were subjected to mechanical stress with the Flexercell FX-5000™ Compression System (Flexcell International) to apply pathophysiological pressure (8–20 kPa, at a frequency of 12 cycles/min) for 48 h; Group SPL, cells treated with 10^−7^ μmol/L spironolactone (Minsheng Pharmacia) under mechanical stress for 48 h; Group SIL, cells transfected with NR3C2-siRNA under mechanical stress for 48 h; Group SCR, cells transfected with scrambled siRNA under mechanical stress for 48 h; Group LY2, cells treated with 5 μmol/L LY294002 (Sigma), inhibitor of PI3K, under mechanical stress for 48 h; Group RAP, cells treated with 5 μmol/L rapamycin (Sigma), an activator of autophagy, under mechanical stress for 48 h.

### Cell adhesion assay

Cell adhesion was performed as previously described [15]. Podocytes (2×10^5^) were seeded in triplicates in the six-well type IV collagen-coated Flexplates. Non-adherent cells were washed off after being exposed to mechanical stress and cells sticking to the bottom were fixed with 4% paraformaldehyde, stained by 0.1% crystal violet. The dye was then washed away and the cellular stain was dissolved in 33% acetic acid. The control cells which grown under identical conditions, but not exposed to stretch were fixed without being washed. Absorbance was quantified with a spectrophotometer at 620 nm optical density. The percentage of podocyte adhesion is presented as the absorbance of experimental groups divided by the absorbance of the control cells (normalized to 1).

### Immunofluorescence analysis

Immediately following strain or static regimens, podocytes were washed with sterile PBS and fixed with 4% paraformaldehyde for 30 min at room temperature. Then they were permeabilized with 0.1% Triton X-100 in 50 mM Tris/HCl (pH 7.4) containing 150 mM NaCl and 1 mg/ml BSA. After that, collagen membranes were cut out of the BioFlex plates and laid on a microscope slide. A coverslip was adhered to the slide with clear nail polish. After washing with PBS, the slides were incubated with anti-LC3 at 4°C (Santa-Cruz Biotechnology) at a 1:200 dilution overnight. Alexa Fluor 488 goat anti-rabbit IgG antibody (Invitrogen) was used as the secondary antibody at a dilution of 1:500 for 30 min. Nuclei of all cells were counterstained with DAPI. Slides were washed and viewed by the laser-confocal microscope (Leica) and analysed by Image Pro Plus software.

### Podocyte transfection

NR3C2-siRNA duplexes were synthesized by Shanghai Genepharma (Genepharma). Sequences were as follows: Forward, 5'-GCCCAGTCGTGTGTATGTTGTCGCGTC-3'; Reverse, 5'-CGACTCTGTAGATCTTCCTGCGCCTTC-3'. When podocytes were grown to 60–70% confluence on 6-hole culture plate, transient transfection was performed with NR3C2-siRNA or control siRNA at a final concentration of 20 nmol/L and 30 nmol/L respectively overnight with Lipofectamine 2000 (Invitrogen) according to the manufacturer's instructions. The transfection efficiency was detected through Real-time PCR or Western blot.

### Western blot analysis

For Western blot analysis, podocytes were lysed in hypotonic lysis buffer (Beyotime), and equal amounts of protein were denatured after heating at 95°C for 5 min and loaded on to an SDS/8% polyacrylamide gel. Separated proteins were subsequently transferred to a nitrocellulose membrane and blocked with 8% nonfat milk at room temperature for 1 h. Membranes with proteins were incubated with primary antibodies at 4°C overnight. The primary anti-MR antibody (1:100, Santa Cruz Biotechnology), anti-human integrin β1 polyclonal antibody (1:400, Bosider), anti-p85-PI3K antibody (1:800, Cell Signaling Technology), anti-p-Akt antibody (ser473, 1:800, Cell Signaling Technology), anti-t-Akt antibody (1:800, Cell Signaling Technology), anti-p-mTOR antibody (ser2448, 1:200, Santa Cruz Biotechnology), anti-Atg5 antibody (1:500, Santa Cruz Biotechnology) and β-actin antibody were used. The second antibodies (horseradish peroxidase conjugate goat anti-rabbit, 1:5000 in blocking solution) were added and incubated for 2 h at 4°C. All blots were developed using Western blotting detection system of enhanced chemiluminescence (Pierce Biotechnology).

### Statistical analysis

SPSS 16.0 software (SPSS Inc., Chicago, IL, USA) was used for the data processing. *P*<0.05 was required for results to be considered statistically significant. Data were presented as mean±S.E.M. and were compared by Student *t* test or ANOVA followed with a post SNK q test as appropriate.

## RESULTS

### Effect of spironolactone on podocyte MR and integrin β1 expression under mechanical stress

**Figure 1:**
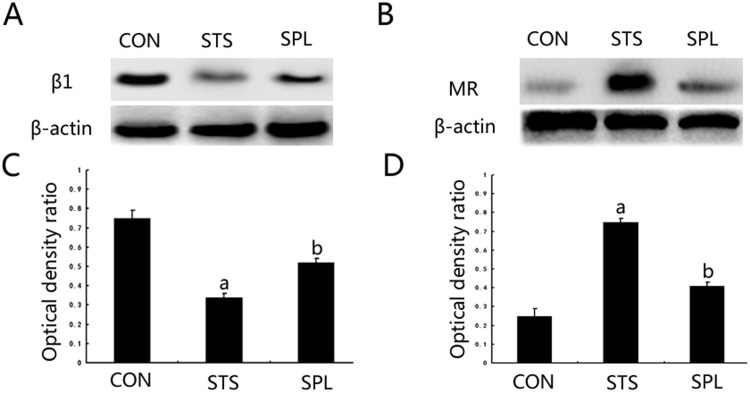
Spironolactone functioned in MR and integrin β1 expression of podocytes exposed to mechanical stress (**A**) Expression changes of protein MR. (**B**) Expression changes of protein integrin β1. (**C** and **D**) The intensities of the bands for MR and integrin β1 protein in (A) and (B) were quantified. a*P*< 0.05 compared with Group CON; ^b^*P*< 0.05 compared with Group STS (*n*=6).

Exposure of podocytes to mechanical stress for 48 h significantly increased cell MR expression and reduced integrin β1 compared with statically cultured podocytes (*P*<0.05). After spironolactone treatment, podocyte MR expression was significantly down-regulated (*P*<0.05) and integrin β1 was significantly up-regulated (*P*<0.05), see Figure 1.

### Influence of mechanical stress on podocyte adhesion capacity and autophagosome formation

**Figure 2:**
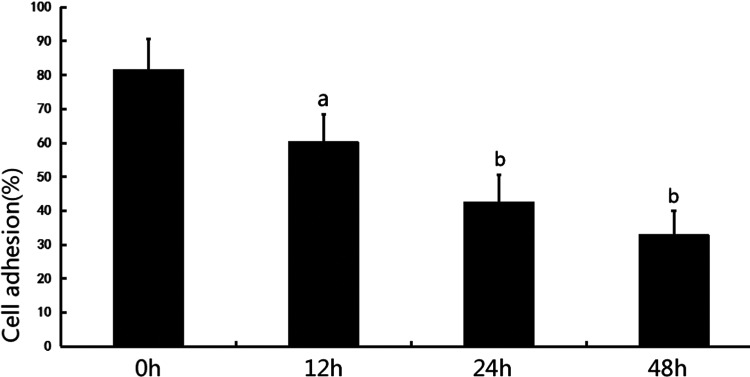
Cell adhesion to collagen type IV was analysed with spectrophotometry for 0 h, 12 h, 24 h and 48 h. a*P*< 0.05 compared with 0 h, ^b^*P*<0.01 compared with 0 h (*n*=6).

An adhesion assay was performed at 0 h, 12 h, 24 h, 48 h and showed a significant reduction in podocytic adhesive capacity at 12 h, 24 h and 48 h compared with 0 h (12 h, 60.3±8.1% compared with 0 h, 81.7±9.0%, *P*<0.05; 24 h, 42.7±8.0% compared with 0 h, 81.7±9.0%, *P*<0.01; 48 h, 32.9±7.1% compared with 0 h, 81.7±9.0%, *P*<0.01), see Figure 2.

**Figure 3:**
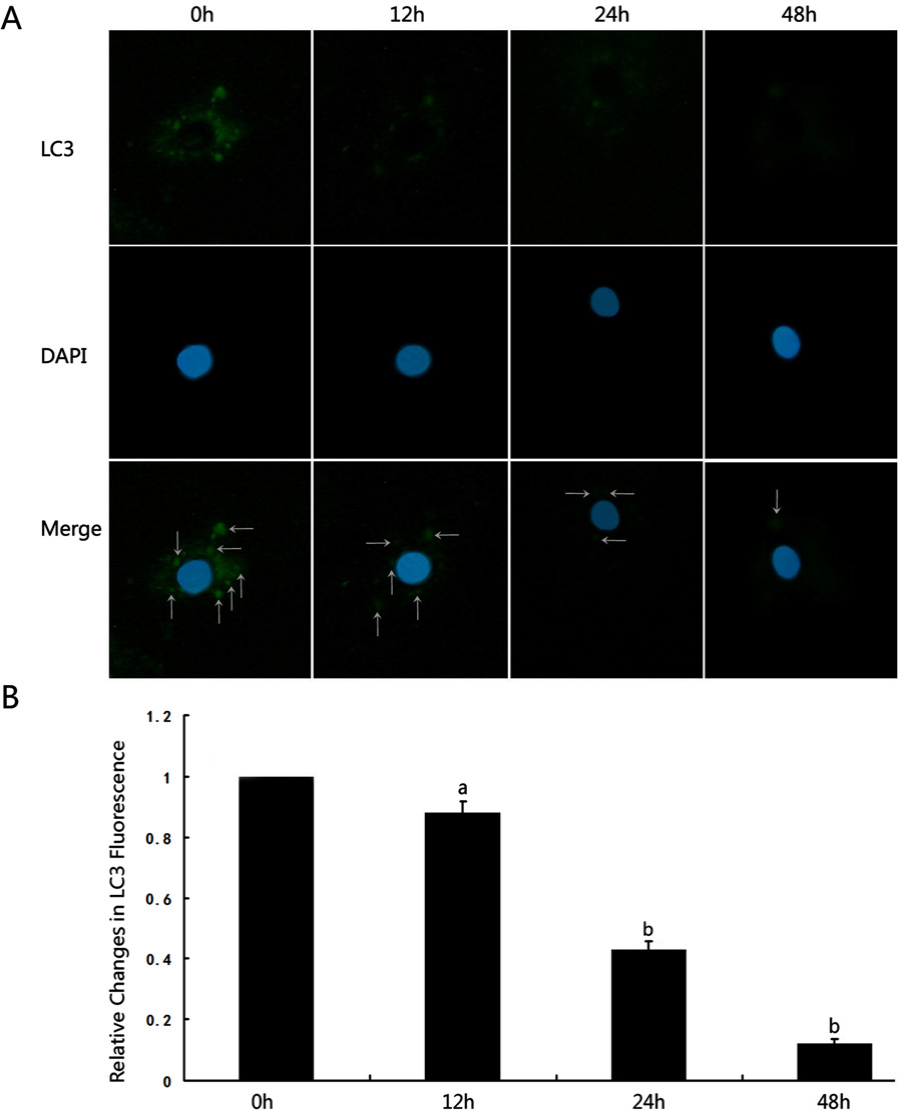
Mechanical stress inhibited autophagy in human podocytes (**A**) LC3-II immunostaining in podocytes under mechanical stress was performed at 0 h, 12 h, 24 h and 48 h visualized by confocal photomicrographs (×1200). (**B**) The representative LC3-II punctate dots quantification was shown along with statistical bar graph analysed by Image Pro Plus software. a*P*> 0.05 compared with 0 h, ^b^*P*<0.05 compared with 0 h (*n*=6).

The LC3-II puncta immunostaining in podocytes was detected in the perinuclear regions at 0 h, 12 h, 24 h, 48 h under mechanical stress. The presence of autophagosomes was observed by the visualization of punctate dots. As shown in Figure 3, the LC3-II punctate dots were remarkable around the perinuclear and cytoplasm regions (indicated by white arrows in merged image) at 0 h in normal podocytes under a confocal microscope. Exposed to mechanical stress, podocytes produced slightly decreased LC3-II punctate dots at 12 h, but no significant difference was observed compared with time point of 0 h (12 h, 0.88±0.04 compared with 0 h, 1, *P*>0.05). At 24 h and 48 h, the LC3-II puncta dots of podocytes were significantly decreased compared with that of 0 h (24 h, 0.43±0.03 compared with 0 h, 1, *P*<0.01; 48 h, 0.12±0.01 compared with 0 h, 1, *P*<0.01), see Figure 3.

### Attenuation of podocytic adhesive capacity damage and autophagy inhibition induced by mechanical stress through spironolactone

**Figure 4:**
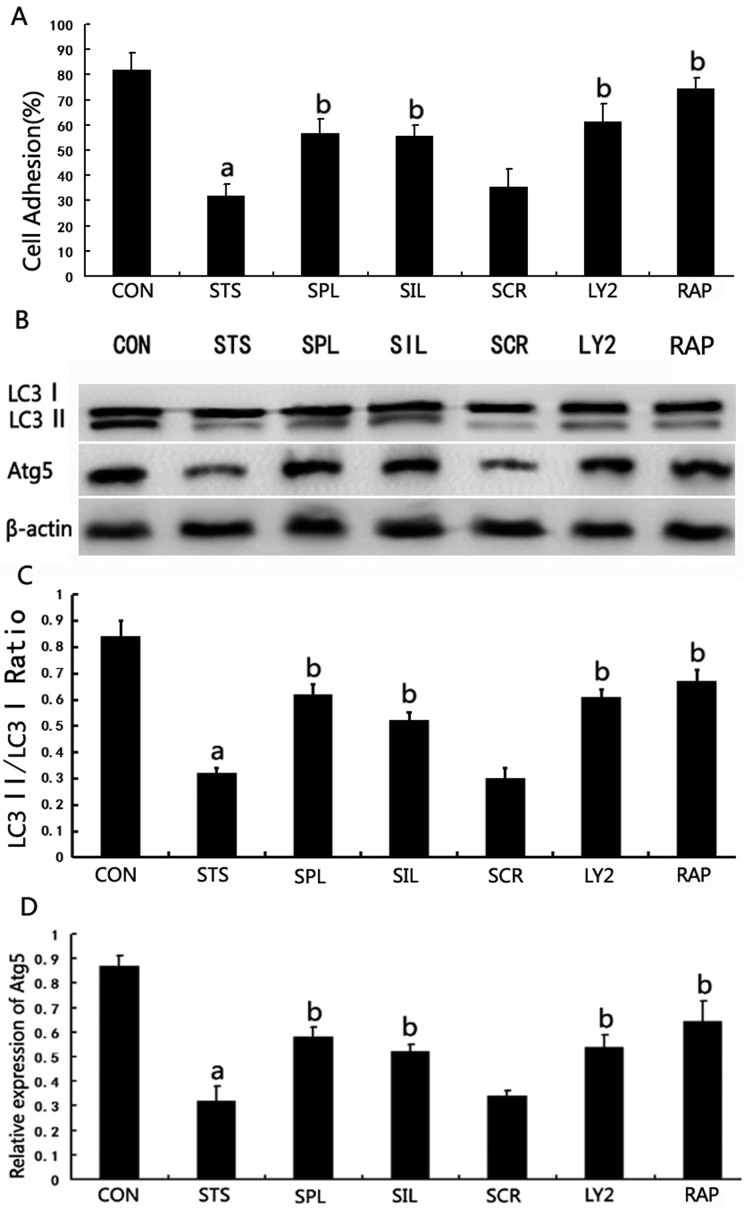
Effect of spironolactone on podocyte adhesion capacity and autophagy induction under mechanical stress (**A**) Effect of spironolactone on podocyte adhesion capacity under mechanical stress. (**B**) Western blot analysis of LC3-II and Atg5 protein expression in each group of podocytes cultured for 48 h. (**C** and **D**) The bar graph shows the densitometric quantification of LC3-II and Atg5 under the indicated conditions. β-Actin served as an internal control. a*P*< 0.05 compared with Group CON; ^b^*P*< 0.05 compared with Group STS (*n*=6).

Podocyte adhesion capacity was significantly increased compared with that of cells exposed to mechanical stress after treatment with spironolactone, LY294002 or rapamycin (*P*<0.05). Moreover, podocytes transfected with NR3C2 siRNA showed remarkably increased adhesion capacity compared with those cells exposed to mechanical stress (*P*<0.05). Podocytes pretreated with scrambled siRNA had no significant changes of adhesion capacity compared with those cells under mechanical stress (*P*>0.05), see Figure 4(A).

To test whether spironolactone induces autophagy in human podocytes, we investigated the expression of LC3-II and Atg5 in treated cells using Western blotting. The results showed that expression of LC3-II and Atg5 in Group STS for 48 h was significantly down-regulated compared with Group CON (*P*<0.05). After spironolactone treatment, LC3-II and Atg5 expression were significantly up-regulated compared with Group STS (*P*<0.05). To test whether spironolactone induces autophagy via blocking MR in podocytes, we silenced podocyte NR3C2 through transfection of NR3C2-siRNA. In Group NR3C2-silenced, LC3-II and Atg5 expression were significantly up-regulated compared with Group STS, whereas there is no statistically significant differences of LC3-II and Atg5 expression in Group SCR. PI3K/AKT/mTOR signalling pathway is involved in the regulation of autophagy. In Group LY294002, LC3-II and Atg5 expression were significantly up-regulated compared with Group STS (*P*<0.05). In Group RAP, LC3-II and Atg5 expression were also significantly up-regulated compared with Group STS (*P*<0.05), see Figures 4(B)–4(D).

### Inhibition of spironolactone on PI3K/AKT/mTOR signalling pathway activation

**Figure 5:**
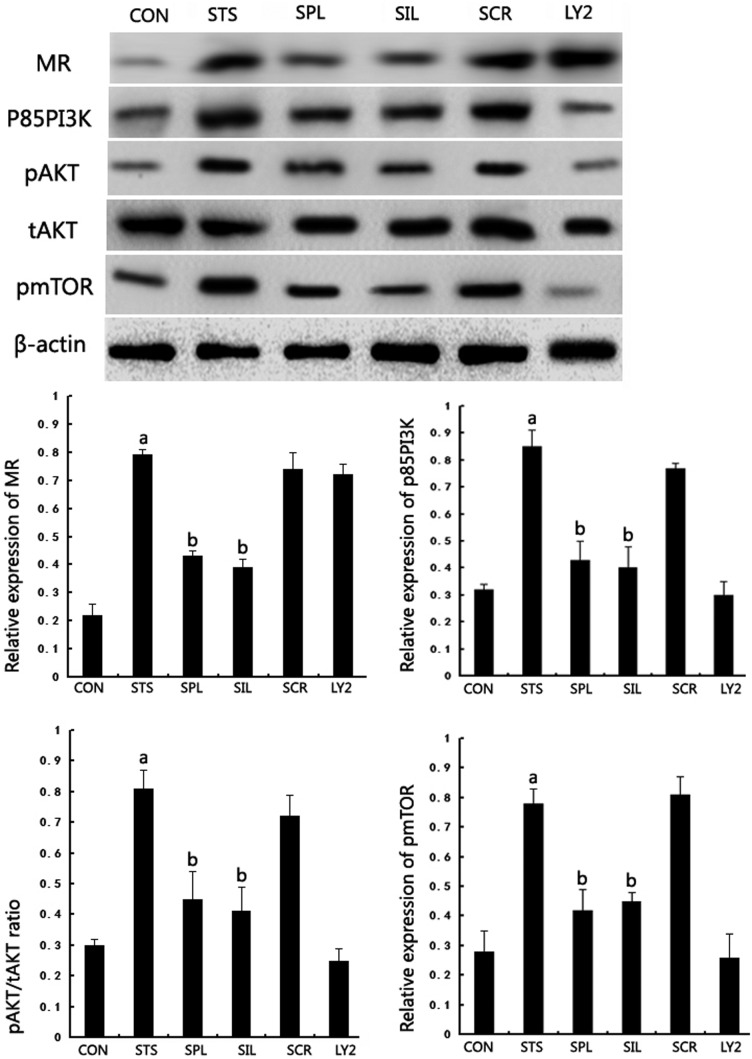
Effect of spironolactone on PI3K/AKT/mTOR signalling pathway related protein expression in podocyte under mechanical stress (**A**) Western blot analysis for MR, p85-PI3K, pAKT, tAKT and pmTOR protein expression in each group of podocytes cultured for 48 h. (**B**) The bar graph shows the densitometric quantification of MR, p85-PI3K, pAKT, tAKT and pmTOR protein expression under the indicated conditions. β-Actin served as an internal control. a*P*< 0.05 compared with Group CON; ^b^*P*< 0.05 compared with Group STS (*n*=6).

A previous study demonstrated the important role of PI3K/AKT/mTOR pathways in negative regulation for autophagy. We next examined whether spironolactone could inhibit the PI3K/AKT/mTOR pathway by analysing the levels of p85-PI3K, pAKT, tAKT and pmTOR using Western blotting. We found that the levels of p85-PI3K, pAKT/tAKT and pmTOR in podocytes under mechanical stress were significantly up-regulated compared with Group CON. In Group SPL, p85-PI3K, pAKT and pmTOR expression were significantly down-regulated compared with Group STS after 48 h (*P*<0.05). In Group NR3C2-silenced, p85-PI3K, pAKT and pmTOR expression were significantly down-regulated compared with Group STS (*P*<0.05). In Group LY294002, p85-PI3K, pAKT and pmTOR expression were significantly down-regulated compared with Group STS (*P*<0.05), whereas MR expression had no significant changes after LY294002 treatment for 48 h (*P*>0.05), see Figure 5.

## DISCUSSION

Our data in the present study reveal that (1) spironolactone could ameliorate podocytic adhesive capacity under mechanical stress via down-regulating protein MR. (2) Podocytes have a certain level of basal autophagy, which may serve as a mechanism for their maintenance of cellular homoeostasis and gradually decrease during mechanical stress adaptation. (3) Spironolactone could attenuate podocytic autophagy inhibition induced by mechanical stress. (4) Spironolactone has beneficial effect on inhibition of PI3K/AKT/mTOR signalling pathway activation in podocytes under mechanical stress. These results suggest that spironolactone could delay the progression of DN by inducing autophagy pathway, specifically targeting PI3K/AKT/mTOR activation.

Podocytes are the main constituents of glomerular filtration membrane and play an important role in the pathogenesis and progression of DN [16]. Recent studies indicate that aldosterone/MR could induce irreversible glomerular podocyte injury and podocyte loss, causing the damages of integrity of filtration barrier and proteinuria [17]. Our previous study reported that spironolactone conferred protection against the podocyte adhesive capacity injury and the proteinuria in streptozotocin-induced diabetic rats [18]. In our present study, we found that podocyte adhesion molecule integrin β1 under mechanical stress were remarkably increased accompanied by MR protein reduction after spironolactone treatment. There might be two reasons why MR is up-regulated in podocytes under mechanical stress. One is that MR is stimulated by aldosterone which is produced by podocytes response to mechanical stress. Another is that MR is up-regulated when podocytes are under mechanical stress. The two common results of MR up-regulation both spring from mechanical stress. The finding of beneficial effect of spironolactone on podocyte prompted us to test the underlying mechanism involved in cellular processes that are regulated by spironolactone under diabetic conditions.

Emerging evidence suggests that autophagy, which is induced by multiple stress factors including hyperglycaemia, oxidative stress, transforming growth factor-β1 (TGF-β1) etc., play an important role in the pathogenesis of DN [19–21]. Haemodynamic changes characterized by increases in high intracapsular pressure, hyperfusion and hyperfiltration have been observed in the early stage of DN [5]. This would lead to an increased mechanical stress on podocytes which as a result, will cause podocyte detachment and apoptosis [22]. Therefore, mechanical stress is an important stimulus that can contribute to podocyte injury in diabetes. However, it is still not clear whether and how autophagy regulation is involved in podocyte injury under mechanical stress in diabetes. In our present study, LC3 immunostaining in podocytes under mechanical stress was performed at 0 h, 12 h, 24 h and 48 h. We detected that podocyte LC3-II puncta was slightly decreased at 12 h and significantly decreased at 24 h and 48 h accompanied by decreased podocytic adhesive capacity, suggesting that autophagy has a renoprotective role and is depressed in podocytes under mechanical stress. To test whether spironolactone induces autophagy in human podocytes, we investigated the expression of LC3-II and Atg5 in treated cells using Western blotting and found that spironolactone significantly up-regulated LC3-II and Atg5 expression. Rapamycin, a classic activator of autophagy, restored podocytic adhesive capacity accompanied by significant up-regulation of LC3-II and Atg5 expression, which suggested that independent activation of autophagy during mechanical stress could reduce podocytic adhesive capacity damage. In the absence of MR in podocytes, LC3-II and Atg5 expression were also significantly up-regulated, suggesting that spironolactone would induce podocyte autophagy under mechanical stress via down-regulation of MR.

**Figure 6:**
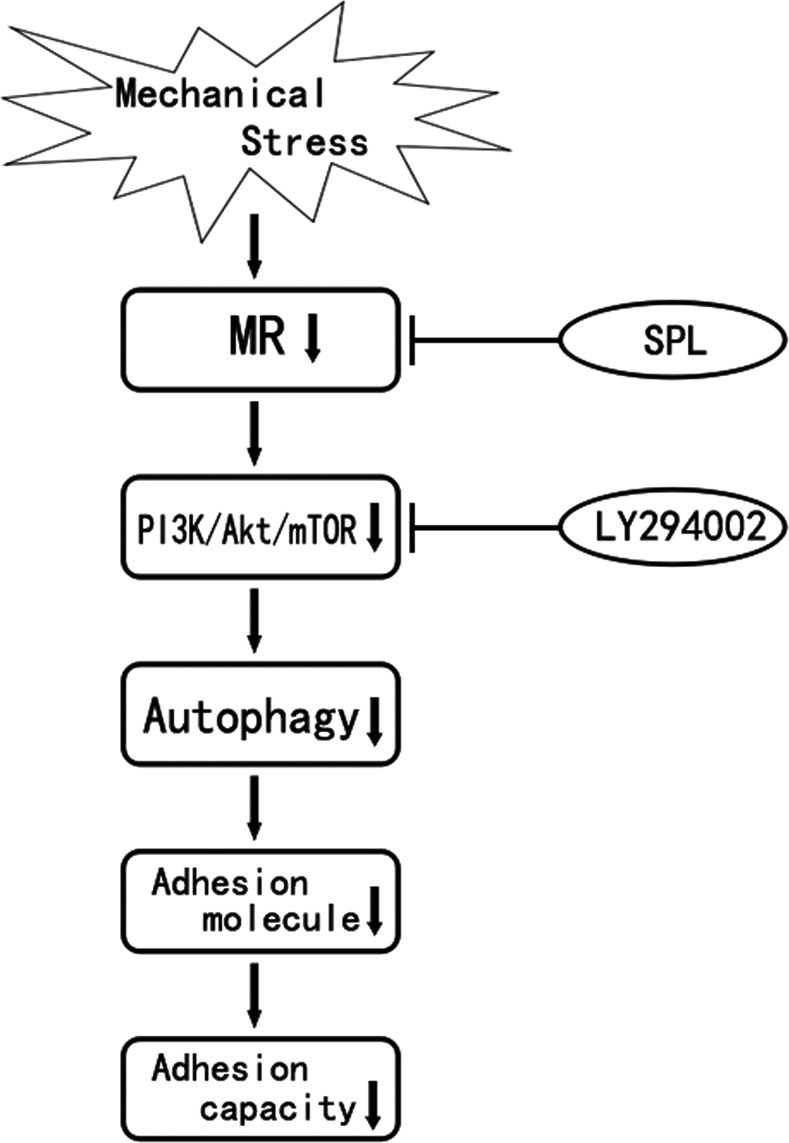
Current hypothesis on the protective mechanism of spironolactone on podocytic adhesive capacity damage under mechanical stress

PI3K/Akt/mTOR signal pathway exists largely in regulating cell growth, apoptosis and other important cellular signal transduction [23,24]. Furthermore, PI3K/Akt/mTOR is the most common regulatory pathway in autophagy response [25]. Previous study showed that Aldo could activate PI3K signalling through MR in human mesangial cells [26]. Hypothetically, we suggested that MR-activated PI3K might be conserved among different species and various cell types. Continuing our observations in the downstream of molecular nature, we examined the harmful effect of mechanical stress on activating the PI3K signalling pathways in podocytes under mechanical stress. In our present study, the levels of p85-PI3K, pAKT/tAKT and pmTOR in podocytes under mechanical stress were significantly up-regulated, whereas after spironolactone treatment, p85-PI3K, pAKT and pmTOR expression were significantly down-regulated. Additionally, LY294002 that inhibits abnormal activation of the PI3K/Akt/mTOR signalling pathway did not alter MR expression, which suggests that spironolactone could induce autophagy in podocytes under mechanical stress through a MR/PI3K/Akt/mTOR-dependent mechanism, see Figure 6.

In conclusion, our data demonstrated for the first time that spironolactone inhibited mechanical-stress-induced podocytic adhesive capacity damage through blocking PI3K/Akt/mTOR pathway and restoring autophagy activity. These results provide novel insights into the renoprotective mechanisms of spironolactone and suggest potential therapeutic strategies for its treatment of DN.

## Abbreviations

DN: diabetic nephropathy
MR: mineralocorticoid receptor
PI3K: phosphatidylinositol 3-kinase
